# Ankle Muscle Activations during Different Foot-Strike Patterns in Running

**DOI:** 10.3390/s21103422

**Published:** 2021-05-14

**Authors:** Jian-Zhi Lin, Wen-Yu Chiu, Wei-Hsun Tai, Yu-Xiang Hong, Chung-Yu Chen

**Affiliations:** 1 Department of Physical Education, National Taiwan University of Sport, Taichung 40404, Taiwan; JZlin@ntus.edu.tw (J.-Z.L.); 102027@ntnu.edu.tw (Y.-X.H.); 2 School of Physical Education, Quanzhou Normal University, Quanzhou 362000, China; dlove520@hotmail.com

**Keywords:** joint motion, landing pattern, biarticular muscle, neuromuscular training

## Abstract

This study analysed the landing performance and muscle activity of athletes in forefoot strike (FFS) and rearfoot strike (RFS) patterns. Ten male college participants were asked to perform two foot strikes patterns, each at a running speed of 6 km/h. Three inertial sensors and five EMG sensors as well as one 24 G accelerometer were synchronised to acquire joint kinematics parameters as well as muscle activation, respectively. In both the FFS and RFS patterns, according to the intraclass correlation coefficient, excellent reliability was found for landing performance and muscle activation. Paired t tests indicated significantly higher ankle plantar flexion in the FFS pattern. Moreover, biceps femoris (BF) and gastrocnemius medialis (GM) activation increased in the pre-stance phase of the FFS compared with that of RFS. The FFS pattern had significantly decreased tibialis anterior (TA) muscle activity compared with the RFS pattern during the pre-stance phase. The results demonstrated that the ankle strategy focused on controlling the foot strike pattern. The influence of the FFS pattern on muscle activity likely indicates that an athlete can increase both BF and GM muscles activity. Altered landing strategy in cases of FFS pattern may contribute both to the running efficiency and muscle activation of the lower extremity. Therefore, neuromuscular training and education are required to enable activation in dynamic running tasks.

## 1. Introduction

Striking one’s feet rapidly and repetitively against the ground is a fundamental running motion in sports. In particular, exceptional running techniques are associated with factors such as forward trunk lean angles [1], stride frequencies and lengths [2], and most importantly, strike patterns [3]. Due to poor running economy between forefoot strike (FFS) and rearfoot strike (RFS), it also affects the appearance of muscle fatigue, which in turn causes lower extremity injuries [4]. Strike patterns can be divided into forefoot strike, midfoot strike (MFS), and rearfoot strike according to the foot strike position [3,5]. Studies have shown that FFS is characterized by a high strike frequency and short stride length [6]. Compared with RFS, FFS allows for shorter contact time [6,7,8], and effective for obtaining a higher sprint speed and step frequency through the greater hip extension and knee flexion velocities during the running phases [3]. The optimal choice between both depends on the sport. For example, sprinters generally choose FFS to increase their starting speed and maintain their maximum speed. However, to achieve these goals, runners must keep their centre of mass at a desirable forward position, which consumes more energy. Therefore, long distance runners typically choose RFS to achieve greater running efficiency. This is because RFS increases running distance instead of running speed and helps runners conserve energy [9,10]. Previous studies have indicated that different foot-strike strategies can affect running economy and the biomechanics of lower extremity joints [11], and that muscle activation is a crucial factor affecting running economy [12,13]. According to these findings, during runs, the lower extremity muscles must have the appropriate joint position, stability, and stiffness to efficiently push the trunk forward. Specifically, muscle activation changes occur to help the human body adapt itself to the dynamic load on its lower extremities during different movements. For example, the muscle activation changes when walking differ from those when running [14,15].

Muscle activation through repetitive tasks originates from foot-floor interaction; such interaction occurs during the landing phase to mitigate the impact of external forces on the foot when landing [15]. Dolenec et al. discovered that when lower extremities strike different ground materials (e.g., asphalt, gravel, and grass), tibialis anterior activation occurs to adjust joint stiffness and maintain a stable foot strike posture [16]. Moreover, in FFS, activation of the gastrocnemius muscle and soleus muscle happens earlier than that in RFS; The gastrocnemius and soleus muscles also play a major role in plantar flexion during the early activated phase. Like the gastrocnemius, it is one of the calf muscles in the back of the leg. It connects to the Achilles tendon at the heel. You need this muscle to push your foot away from the ground, so the plantar flexion muscle to provide FFS with higher levels of muscle hypertrophy [17] and form an isometric or lengthening contraction [18,19].

The aforementioned studies have highlighted the consequential effect of lower extremity neuromuscular activation on the running economy. For example, previous studies reported that different foot strike strategies can affect the running economy and the biomechanics of lower extremity joints [11], and that muscle activation is a crucial factor affecting the running economy [12,13]. Running economy can be quantified as the steady-state oxygen consumption during steady-state rate [20]. There is evidence that running economy is worse when running between FFS and RFS patterns [11]. According to these findings, during runs, the lower-extremity muscles must have the appropriate joint position, stability, and stiffness to efficiently push the trunk forward. However, the effects of the characteristics of neuromuscular activation in different foot strike patterns on the benefits of running techniques merit further exploration. Accordingly, the present study compared the joint kinematics and muscle activation in the pre-landing and stance phases between different strike movements. Next, this study identified how lower-extremity activation contributed to running technique development.

## 2. Materials and Methods

### 2.1. Participants

Ten division I male physical education students volunteered for the study (age: 21.7 ± 1.9 years; height: 1.70 ± 0.05 m; mass: 65.1. ± 5.5 kg). All participants had completed all experimental running conditions, and were free of any previous lower extremity injury at the time of testing. All participants were required to run at least 10 km per week and all were comfortable running on the treadmill for this study. The participant inclusion criteria included (1) no complaints of back, hip, knee, or ankle pain, (2) resumption of all pre-injury activities without limitation for at least 12 months before testing, and (3) no musculoskeletal trauma or neurosurgery within the previous six months. We excluded all participants that had sustained injury to any of their lower extremities within the previous year. In this study, the research was explained to every participant, and written consent was provided before participating in the study. The study was approved by the Ethics Committee of Antai Medical Care Cooperation Hospital (18-104-B).

### 2.2. Instrumentation

**Figure 1:**
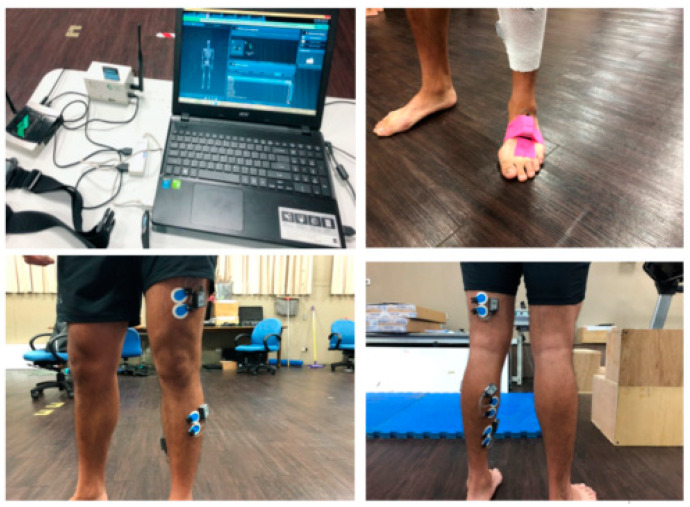
Full set-up position of IMU and EMG sensors.

Three IMU sensors (200 Hz; Myomotion, Noraxon Inc., Scottsdale, AZ, USA) were used for measurement of lower extremity joint kinematics in the sagittal plane. IMU data were recorded using a wireless inertial measurement system consisting of miniature IMU sensors securely mounted unilaterally to the shank (dominant leg), thigh (dominant leg), and pelvis. A Noraxon system with a 24G wireless accelerometer (1500 Hz) and five wireless electromyography sensors (1500 Hz) were used to define the initial foot contact and to collect muscle activity (Figure 1). According to SENIAM recommendations, double differentiated surface electrodes were placed in the direction of muscle fibres on shaved cleaned skin: (1) rectus femoris (RF), (2) biceps femoris (BF), (3) tibialis anterior (TA), (4) gastrocnemius medialis (GM), (5) soleus (SO) [21]. The maximum voluntary isometric contraction (MVIC) values were obtained to normalise some of the studied variables [22]. The ankle and knee muscle groups’s MVICs were performed using the BIODEX dynamometer (Biodex Systems 4 Pro, Biodex Inc., Shirley, New York, NY, USA). Each set of three MVICs was followed by at least 3 min rest. To minimize the effects of fatigue, at least 5 min was allowed between each MVIC group [23].

### 2.3. Data Collection

This protocol ensured that stable kinematics and muscle activity data could be collected after the 1–20 cycles necessary for the neuromuscular system to adapt to drastically altered task mechanics. Participants were asked to run on a treadmill at 6 km/h and perform two foot strike patterns (i.e., FFS and RFS patterns) in a random order. They performed a 5-min barefoot warm-up with a treadmill (Horizon Paragon 4, Johnson Inc., Taichung, TW), run at a free speed to familiarise themselves with running without shoes. A wireless accelerometer was fixed to the foot instep of the dominant leg prior to the running task. Every trial for the foot strike pattern lasted >30 s. The consecutive trials with a rest period of >1 min [13], and all the foot strike patterns were confirmed visually. Participants were allowed to make additional attempts until each strike pattern was completed with three successful trials. There were three consecutive FFS and then three RFS conducted randomly between FFA and RFS patterns. Each participant’s dominant leg was defined as the limb that would be used to kick a ball [24].

### 2.4. Data Analysis

**Figure 2:**
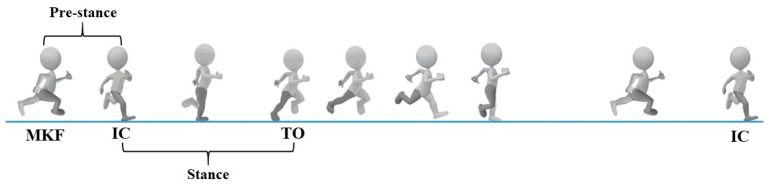
The phases of the running gait cycle. Note: MKF indicates maximum angle of knee flexion; IC indicates initial contact; TO indicates take-off; Pre-stance phase is defined as MKF to IC; Stance phase is defined as IC to maximum velocity of knee extension.

The phase definition of this study is based on the running gait cycle. It is hoped that the simplest research equipment will be used for the defined phase, and the innovative staging method will be used to define the pre- and post-landing phase of this study simply and quickly, so as to formulate a new experimental procedure. The running gait cycle was further divided into two phases: pre-stance and stance (Figure 2). The pre-stance phase was defined as the swing phase, with the maximum angle of knee flexion occurring prior to initial contact. The stance phase was defined as the initial contact with the force plate prior to maximum velocity of knee extension. Initial contact was defined as the peak value during the landing phase in accelerometer signals of the vertical axis. Maximum push-off velocity was defined from initial contact to maximum velocity of knee extension. Kinematics and EMG data were processed using Noraxon 3.8.6 software. The IMU data were filtered using a cut-off frequency of 6 Hz with a fourth-order zero-lag Butterworth digital filter. The EMG process used the 10 to 500 Hz band-pass filter. The root mean square (RMS) algorithm was calculated using a 20-sample moving average. [25].

### 2.5. Statistical Analysis

Statistical analysis (SPSS 18, Inc., Chicago, IL, USA) was performed, and descriptive statistics (mean and standard deviation) were used to determine the characteristics of the participants. Paired sample *t*-test was used to compare initial contact of ankle angle, the maximum push-off velocity of knee extension, and muscle activation in the FFS and RFS strike patterns. The intraclass correlation coefficients (ICCs) were calculated. To assess the consistency and test–retest reliability of the measurement of joint pattern and muscle activation in five strikes of each pattern, the effect size (ES) was evaluated according to the method of Cohen [26]. The level of significance was set at *p* < 0.05.

## 3. Results

### 3.1. The Intraclass Correlation Coefficient of Participants

The reliability of the landing performance and muscle activity was generally good. Within an FFS pattern, initial contact angle, maximum push-off velocity, and muscle activation all showed good reliability (Table 1). Specifically, the RFS strike pattern also had excellent reliability according to ICC data, mostly greater than 0.900 (Table 1).

### 3.2. Kinematics Data

**Figure 3:**
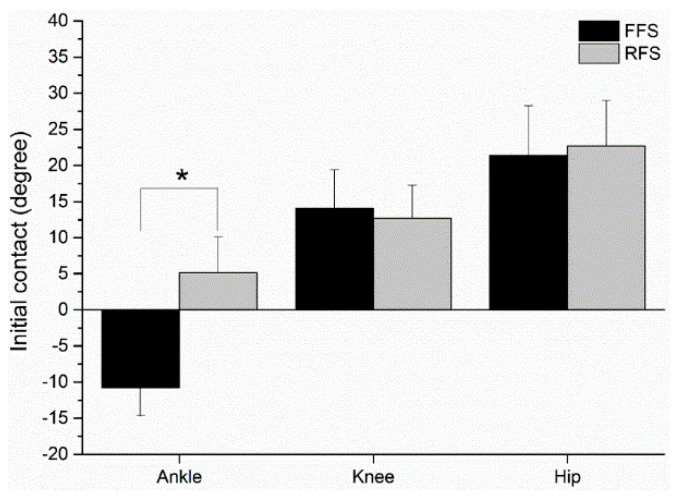
FFS and RFS patterns of sagittal plane at initial contact. Note: * indicate *p* < 0.05; FFS = forefoot strike pattern; RFS = rearfoot strike pattern; negative values (−) indicate ankle plantar flexion; positive values (+) indicate ankle dorsiflexion.

**Figure 4:**
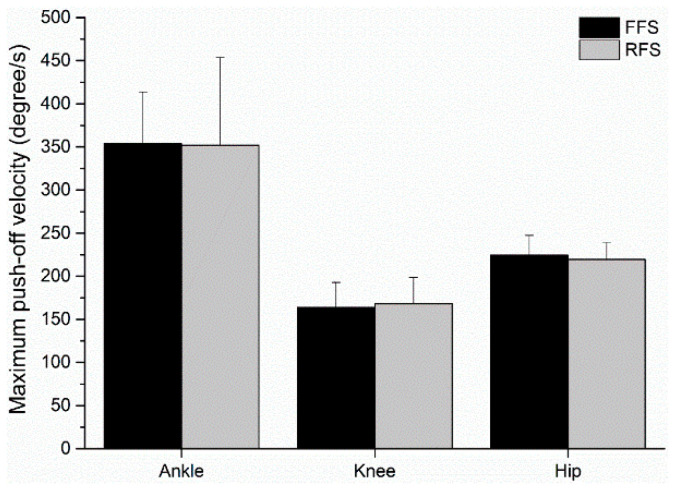
Maximum velocity of push-off of FFS and RFS pattern during stance phase. Note: FFS = forefoot strike pattern; RFS = rearfoot strike pattern.

At the initial contact of the strike pattern, compared to the FFS pattern, the RFS pattern had a significantly greater ankle flexion, *t*(9) = 4.753, *p* = 0.001, *d* = 1.50, 95% CI [8.34, 23.48] (Figure 3). Additionally, the knee and hip joint angle indicated no significant difference between the FFS and RFS pattern at the initial contact (*ps* > 0.05). For the ankle, knee and hip maximum velocity at push-off, no significant differences were found between the FFS and RFS patterns (*ps* > 0.05; Figure 4).

### 3.3. EMG Activities

**Figure 5:**
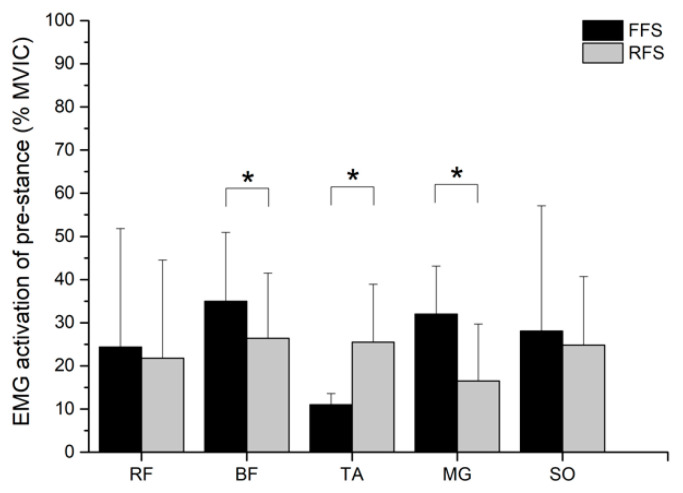
Muscle activity of FFS and RFS pattern during the pre-stance phase. Note: * indicates *p* < 0.05; FFS = forefoot strike pattern; RFS = rearfoot strike pattern; RF = rectus femoris; BF = biceps femoris; TA = tibialis anterior; GM = gastrocnemius medialis; SO = soleus.

**Figure 6:**
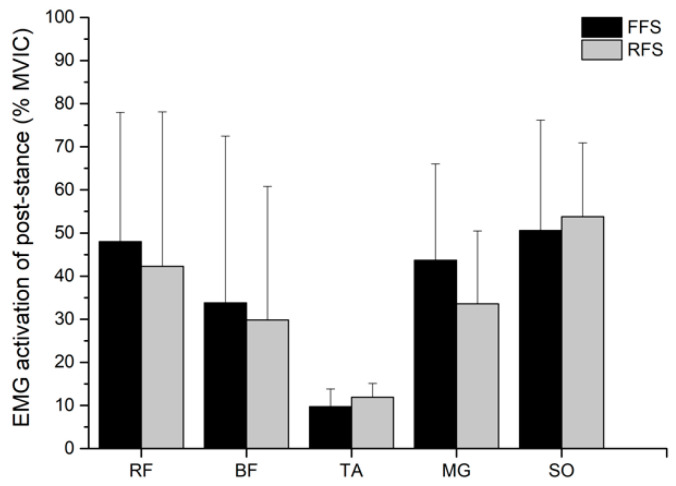
Muscle activity of FFS and RFS pattern during stance phase. Note: FFS = forefoot strike patter; RFS = rearfoot strike pattern; RF = rectus femoris; BF = biceps femoris; TA = tibialis anterior; GM = gastrocnemius medialis; SO = soleus.

The BF muscle demonstrated an activation increase in the pre-stance phase of the FFS pattern compared to the RFS pattern, *t*(9) = −3.848, *p* = 0.004, *d* = −1.22, 95% CI [−13.53, −3.51], and the TA muscle increased in the pre-stance phase of RFS pattern compared to in the FFS pattern, *t*(9) = 3.652, *p* = 0.005, *d* = 1.15, 95% CI [5.53, 23.55]. During the pre-stance phase, the FFS pattern showed significantly greater MG muscle compared to the RFS pattern, *t*(9) = −3.917, *p* = 0.004, *d* = −1.24, 95% CI [−24.37, −6.53] (Figure 5). No significant difference in muscle activity was found between the FFS and RFS pattern during the stance phase (*ps* > 0.05; Figure 6).

## 4. Discussion

The purpose of this study was to clarify whether different foot strike patterns are as-sociated with varying landing performance and muscle activity in athletes. The results demonstrated significantly high internal consistency in joint motion (ICC above 0.900) and muscle activity (ICC above 0.800) for five strikes in each pattern (Table 1). Because running movement is a repetitive task, higher consistency was found for lower extremity joint and muscles activity with the loading rate of impact force [14]. A previous study revealed that lower extremity joints and muscles must provide suitable joint position, stiffness, and stability, as well as propulsion to move the upper body during running. Thus, in FFS and RFS patterns, changes in joint motion and muscle activity are expected in response to different loadings of the lower extremity during running [14,15]. However, during regular running speed, the lower extremity maintains similar joint angles and muscle activity. Therefore, high consistency in the running pattern reduces energy consumption and maintains running economy [11,13].

Our results confirm that the characteristics of different foot strike patterns were successfully manipulated in this study. At the initial landing contact, compared with the FFS pattern, the RFS pattern had a significantly higher dorsiflexion angle (Figure 3). Different foot strike patterns are currently recognized to affect running performance and muscle activity [3]. The ankle helps maintain body stability [27]. In this study, no difference was observed between the knee and hip joint, but a larger ankle motion was found in the FFS pattern than in the RFS pattern of running (Figure 3). According to our results, the ankle strategy focused on controlling the foot strike pattern. During initial contact, the FFS pattern used an ankle plantar flexed position to produce higher GM activity [5]. Conversely, the RFS pattern used ankle dorsiflexion during running, which may contribute to activation of the TA muscle [8,28]. Our findings indicate that higher EMG amplitude of the BF and GM and lower EMG amplitude of the TA were present in the FFS pattern than in the RFS pattern (Figure 5). In this study, the participants with FFS running would activate muscles to a greater degree than the RFS pattern. The results of this study agree with our hypothesis. These findings are similar to those of previous studies [18,28,29]. Some studies have suggested that muscle activity is an important mechanic that influences the running economy [12,13]. Seki et al. indicated that while altering muscle activation during running, the determinant factors of energy cost focus on running economy. Electromyography is also a factor that is important to consider when comparing different strike patterns and energy costs [30]. In comparison, EMG could be a method for detecting muscle activation, and it influences energy costs, and could be used to evaluate the activities of bi-articular muscle and co-activation of agonists and antagonists [31]. Our data demonstrated that, compared with RFS running, FFS running was associated with moderate but significantly higher activation of the GM and BF. Biarticular muscles are muscles that cross two joints, such as the GM muscle, which crosses both the ankle and knee, and the other hamstring muscle, which cross both the knee and the hip. Moreover, biarticular muscles can also use mechanical effects between ankle and knee joints (or knee and hip joints). However, the magnitude and direction of this are dependent on anatomy, joint angle, and muscle activity level [32].

For the FFS pattern, the biarticular muscles are a crucial consideration when landing behaviours have potential benefits or positive consequences. When performing a specific strike pattern, this will directly affect the running speed depending on the loading conditions of the muscle [32]. During the pre-stance phase, the FFS pattern was associated with significantly higher activation in the GM (Figure 5). The GM is essential for controlling the dynamic stability of the ankle and knee joints, while at the same time being the first muscle to respond to sudden ankle stress [33]. The stretch-shortening cycles (SSC) could store a large amount of elastic energy prior to shortening. There are a lot of benefits to ankle plantar flexors, which are advantageous for power output and driving force supply during the descending phase [32,34]. Therefore, the major findings of the present study indicate higher EMG amplitude of the BF and GM and lower EMG amplitude of the TA in the FFS pattern. Sawicki et al. reported that due to differences in the muscle-tendon structure and the proximo-distal joint, lower extremity joints have different muscle activation efficiencies. [35]. On the other hand, a part of mechanical energy is probably stored in the muscles from recoil as elastic energy [36], which could possibly increase the efficiency of the FFS pattern. In this study, compared with the knee and hip joints, the ankle joint has greater efficiency for store and return elastic energy [30,35]. These findings would be useful for providing athletes who use the FFS pattern with the capacity to train the muscle around the ankle joint. This would help in improving the running economy in level running.

During the pre-stance phase, FFS had a significantly greater EMG amplitude of BF compared to RFS (Figure 5). At the initial contact, the BF muscle provided hip extensors and knee flexors to counteract the effect of ground reaction force, which also plays an important role in joint stability, specifically for ACL. For FFS runners, the hamstring muscles had a significant protective effect on the knee joint. During high-speed running, strong and powerful hamstring muscles could help provide deceleration for the knee, such as by limiting forward movement of the tibia and reducing ACL injury [37]. The FFS pattern used ankle plantar, which flexed when contacting the ground, this could cause a longer period at the ankle, allowing force attenuation of landings and thus relieving the knee joint [5,28]. The activation of the BF has a direct relationship with hip and knee joint motion. When the lower extremity is extended at the knee, the hip flexes, and BF is insufficiently activated, because increased activation of the BF is able to help the knee produce a flexion effect, which is good for knee and hip joints during running. A previous study revealed that participants who used the FFS pattern were able to achieve a greater peak ankle plantarflexion moment and lower peak knee extensor moment, estimated peak quadriceps force, and patellofemoral joint stress [11,38,39]. Therefore, if the quadriceps muscle is activated to too great a degree, it will result in a high risk of ACL, and the activity of BF will help balance the relationship between Q:H ratio [40]. When the lower extremity lands on the ground, knee movement is limited by the ground, and the increased activation of the BF will be transferred to hip extension, which facilitates the trunk forward movement. At the same time, the BF activation may affect reduction of knee extensor moments, and the decrease the risk of anterior cruciate ligament. As mentioned, the FFS strategy can contribute to increasing BF and GM muscle activity following short-term adoption. We suggest that the FFS strategy, adapted for daily training, increases neuromuscular activation and provides effective training of the hamstring muscle.

## 5. Conclusions

During the landing phase, the ankle strategy is essential in repetitive tasks, such as running. The FFS and RFS strike patterns lead to different muscle activities in the lower extremities. For muscle activation, the FFS pattern could help to increase the muscle activation of GM and BF. The GM provides a sufficient ankle push-off and knee lift to increase running efficiency, especially accelerated running. Appropriate BF activation provides a balance of the QH ratio and reduces the risk of ACL injury. Therefore, the modification of running techniques with FFS patterns may increase running performance and neuromuscular training.

## Figures and Tables

**Table 1:** The intraclass correlation coefficient of joint kinematics and muscle activation during stance phase.

Parameters	FFS		RFS	
	Pre-Stance	Stance	Pre-Stance	Stance
Initial contact angle				
Hip	0.989		0.989	
Knee	0.975		0.969	
Ankle	0.983		0.981	
Maximum push-off velocity				
Hip	0.906		0.970	
Knee	0.922		0.928	
Ankle	0.988		0.967	
Muscle activation				
RF	0.976	0.995	0.990	0.978
TA	0.815	0.968	0.978	0.915
BF	0.986	0.989	0.964	0.993
GM	0.929	0.986	0.942	0.978
SO	0.996	0.970	0.984	0.948

Note: FFS = forefoot strike pattern; RFS = rearfoot strike pattern; RF = rectus femoris; BF = biceps femoris; TA = tibialis anterior; GM = gastrocnemius medialis; SO = soleus.

## Data Availability

Data is contained within the article.
